# The genome of the largest bony fish, ocean sunfish (*Mola mola*), provides insights into its fast growth rate

**DOI:** 10.1186/s13742-016-0144-3

**Published:** 2016-09-09

**Authors:** Hailin Pan, Hao Yu, Vydianathan Ravi, Cai Li, Alison P. Lee, Michelle M. Lian, Boon-Hui Tay, Sydney Brenner, Jian Wang, Huanming Yang, Guojie Zhang, Byrappa Venkatesh

**Affiliations:** 1 State Key Laboratory of Genetic Resources and Evolution, Kunming Institute of Zoology, Chinese Academy of Sciences, Kunming, China; 2 China National Genebank, BGI-Shenzhen, Shenzhen, China; 3 Comparative Genomics Laboratory, Institute of Molecular and Cell Biology, A*STAR, Biopolis, Singapore, 138673 Singapore; 4 BGI-Shenzhen, Shenzhen, 518083 China; 5 James D Watson Institute of Genome Sciences, Hangzhou, 310058 China; 6 Centre for Social Evolution, Department of Biology, University of Copenhagen, Copenhagen, Denmark; 7 Department of Paediatrics, Yong Loo Lin School of Medicine, National University of Singapore, Singapore, 119228 Singapore

**Keywords:** Ocean sunfish, *Mola mola*, Growth rate, Body size, Cartilaginous skeleton, Positive selection

## Abstract

**Background:**

The ocean sunfish (*Mola mola*), which can grow up to a length of 2.7 m and weigh 2.3 tons, is the world’s largest bony fish. It has an extremely fast growth rate and its endoskeleton is mainly composed of cartilage. Another unique feature of the sunfish is its lack of a caudal fin, which is replaced by a broad and stiff lobe that results in the characteristic truncated appearance of the fish.

**Results:**

To gain insights into the genomic basis of these phenotypic traits, we sequenced the sunfish genome and performed a comparative analysis with other teleost genomes. Several sunfish genes involved in the growth hormone and insulin-like growth factor 1 (GH/IGF1) axis signalling pathway were found to be under positive selection or accelerated evolution, which might explain its fast growth rate and large body size. A number of genes associated with the extracellular matrix, some of which are involved in the regulation of bone and cartilage development, have also undergone positive selection or accelerated evolution. A comparison of the sunfish genome with that of the pufferfish (fugu), which has a caudal fin, revealed that the sunfish contains more homeobox (Hox) genes although both genomes contain seven Hox clusters. Thus, caudal fin loss in sunfish is not associated with the loss of a specific Hox gene.

**Conclusions:**

Our analyses provide insights into the molecular basis of the fast growth rate and large size of the ocean sunfish. The high-quality genome assembly generated in this study should facilitate further studies of this ‘natural mutant’.

**Electronic supplementary material:**

The online version of this article (doi:10.1186/s13742-016-0144-3) contains supplementary material, which is available to authorized users.

## Background

The ocean sunfish *Mola mola* (Family Molidae; Order Tetraodontiformes), is the heaviest known bony fish with the largest recorded specimen measuring 2.7 m in length and weighing 2.3 tons [1]. It is widely distributed in tropical and temperate sea zones, such as the Mediterranean, the North and South Atlantic, the Gulf of Mexico, and the East and West Pacific [1]. The ocean sunfish (hereafter referred to as ‘sunfish’) has several unique characteristics compared with other tetraodontiform fishes such as pufferfish, boxfish and triggerfish. The sunfish passes through two distinct larval stages during its transition to adult stage: the first is a typical pufferfish-like stage in which the fry resembles a miniature pufferfish possessing large pectoral fins, a tail fin and body spines; and the second is a highly transformed stage during which the tail is completely absorbed [2]. The most notable characteristics of sunfish are its fast growth rate and large body size. A captive sunfish gained approximately 400 kg in just 15 months with an average growth rate of 0.82 kg/day [3], which is in stark contrast to the typical growth rate of other teleost fishes (0.02 to 0.49 kg/day) [3, 4]. Furthermore, although sunfish is a bony fish (Osteichthyes), its endoskeleton is mainly composed of cartilage [5]. This lighter cartilaginous skeleton, together with its thick layer of low-density, subcutaneous, gelatinous tissue, may contribute to the buoyancy of this enormous fish [6]. Another unique characteristic of the sunfish is the degeneration of the vertebral column resulting in the loss of the caudal fin, which is instead replaced by a broad and stiff lobe called the ‘clavus’ [1]. As a result, the lateralis muscles of the sunfish insert upon the deep muscles of the anal and dorsal fins that function as the main locomotory organs. Because of this morphological change, the sunfish swims in a peculiar manner. Unlike the drag-based swimming of most bony fishes, the sunfish swims by moving its dorsal and anal fins synchronously to generate a lift-based thrust that resembles the flight of a bird [6]. This unusual swimming mode may involve modifications in the nervous system that controls locomotion. Indeed, anatomical studies have shown that the peripheral nervous system of the sunfish differs from other Tetraodontiformes fishes [7]. The sunfish is also the most fecund vertebrate with a 137 cm female producing an estimated 300 million eggs [1].

Although the behavioural and ecological features of the sunfish have been studied widely, the genomic basis of its unique phenotype remains unexplored. In this study, we have sequenced the genome of the sunfish, and performed comparative genomic analyses with several other fish genomes. We analyzed genes and pathways associated with the regulation of growth and found notable changes in several genes in the growth hormone and insulin-like growth factor 1 (GH/IGF1) axis, an important pathway for regulating growth. In addition, many genes that are associated with the extracellular matrix (ECM) exhibit elevated *dN/dS* values, which may be related to the evolutionary changes in the endoskeleton of the sunfish.

## Results

### Genome assembly and annotation

**Fig. 1:**
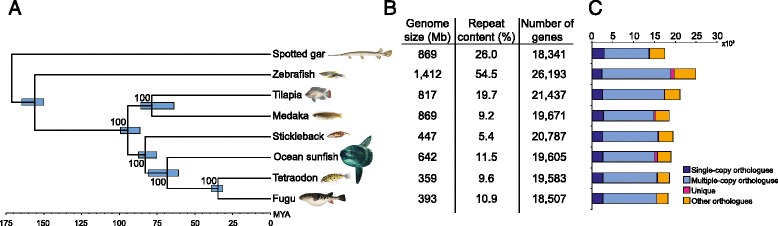
Divergence times and genome statistics of representative ray-finned fishes. **a** Divergence times of representative ray-finned fishes estimated using the topology obtained from the phylogenomic analysis (see Methods). The blue bars on ancestral nodes indicate the 95 % confidence intervals of divergence time estimates (MYA, million years ago). Numbers on each node represent bootstrap support values. **b** Genome statistics and (**c**) distribution of different types of orthologues in representative ray-finned fishes. The repetitive content of sunfish, fugu, *Tetraodon*, medaka, zebrafish, tilapia and stickleback were estimated in the present study (see Methods) whereas that for spotted gar is from Braasch et al. [79]

A total of 98.22 Gb raw reads were generated by sequencing eight paired-end libraries with insert sizes ranging from 170 to 40 kb (Additional file 1: Tables S1 and S2) using the Illumina HiSeq 2000 platform, resulting in ~96X coverage of the k-mer estimated genome size of sunfish (see Methods and Additional file 1: Tables S2 and S3). The reads were assembled using SOAPdenovo [8] to generate an assembly spanning 642 Mb of an estimated 730 Mb genome size (see Methods), with a contig N50 length of 20 kb and a scaffold N50 length of 9 Mb (see Additional file 1: Tables S3 and S4). The sunfish genome comprises approximately 11 % repetitive sequences (transposable elements, tandem repeats and simple-sequence repeats; see Additional file 1: Tables S5 and S6), which is comparable to the repeat content of the fugu genome (Fig. 1 and Additional file 1: Tables S5 and S6). Using homology-based and *de novo* annotation methods, we predicted 19,605 protein-coding genes in the sunfish assembly (Fig. 1; see Methods). Around 95 % of the predicted sunfish protein sequences show similarity to protein sequences in public databases. Using a genome-wide set of 1690 one-to-one ray-finned fish orthologues (identified using a combination Ensembl Biomart data and InParanoid analysis) in sunfish and seven other ray-finned fishes (fugu, *Tetraodon*, stickleback, medaka, tilapia, zebrafish and spotted gar), we reconstructed a phylogenetic tree and estimated the divergence times of various fish lineages using MCMCtree [9] (see Methods). Our analysis (Fig. 1) suggests that sunfish (Molidae) and pufferfishes (Tetraodontidae) separated approximately 68 million years ago (mya; confidence interval 60.8 to 80.8 mya), which corroborates the results of other recent studies that are based on smaller datasets [10, 11].

### Population size history

We identified approximately 489,800 heterozygous single nucleotide polymorphisms (SNPs) in the genome assembly of the sunfish and estimated the heterozygosity to be 0.78 × 10^−3^, which is lower than other marine fishes such as Atlantic cod (2.09 × 10^−3^) and stickleback (1.43 × 10^−3^) [12]. Based on the identified heterozygous sites, we ran the pairwise sequentially Markovian coalescent (PSMC) model [13] to infer the historical changes in the effective population size (N_e_) of sunfish. The PSMC analysis suggests that there was an increasing trend of N_e_ from ~3 to ~0.9 mya (Additional file 1: Figure S1). Around 2.15 mya, a large asteroid (more than 1 km in diameter) is thought to have fallen into the Southern Ocean in the Eltanin Fault zone and generated a super-tsunami that resulted in a large-scale marine extinction [14]. This event might have released more habitats that enabled sunfish to expand its population size. Around 0.9 mya, the N_e_ stopped expanding and began to decline slightly, which could be related to the mid-Pleistocene climate transition (MPT, ~1.2-0.55 mya, Additional file 1: Figure S1) [15]. The MPT period was accompanied by the extinction of many marine species such as *Stilostomellidae* and *Pleurostomellidae* [16]. We also found a N_e_ peak around 150 thousand years ago (kya), followed by a rapid decrease of N_e_. However, the bootstrap support for these estimates is rather weak (Additional file 1: Figure S1).

### Positively selected and fast-evolving genes

**Fig. 2:**
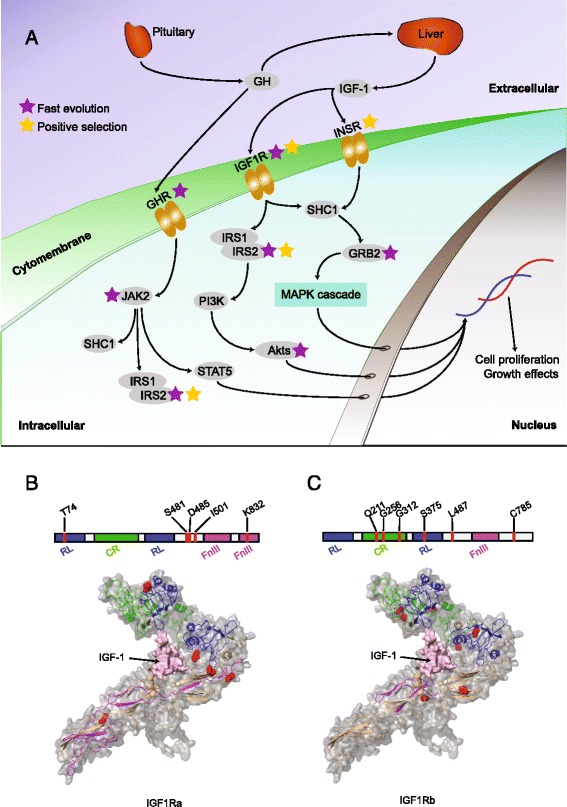
Fast-evolving and positively selected genes in the GH/IGF1 axis. **a** Schematic representation of GH/IGF-1 signalling, adapted from [80]. Arrows denote the direction of signal transduction, whereas the grey ellipse represents an enzyme or a cytokine. Purple stars indicate genes exhibiting elevated *dN/dS* (fast evolution), whereas the yellow stars indicate positive selection. **b** 3D structure of IGF1Ra and (**c**) IGF1Rb monomers as predicted by the SWISS-MODEL Workspace [81]. The bar above the 3D structure indicates the structural domains. Red lines in the bar and the red atom balls in the 3D structure represent the positively selected sites. Domains in the 3D structure are coloured according to the colour scheme on the bar. The pink-surface model in the centre of the IGF1R structure is IGF-1. Akt, protein kinase B; CR, furin-like cysteine rich region (PF00757); FnIII, fibronectin type III domain (PF00041); GH, growth hormone; GHR, growth hormone receptor; GRB2, Growth factor receptor-bound protein 2; IGF-1, Insulin-like growth factor 1; IGF1R, Insulin-like growth factor 1 receptor; INSR, Insulin receptor; IRS, Insulin receptor substrate; JAK2, Janus kinase 2; MAPK, Mitogen-activated protein kinase; PI3K, phosphoinositide 3-kinase; RL, receptor L domain (PF01030); SHC1, SHC-transforming protein 1; STAT5, signal transducer and activator of transcription 5

Using a set of 10,660 one-to-one teleost homologues (determined by reciprocal best BLASTP hit with an E-value cutoff of 1e-5) from five teleost species (sunfish, fugu, *Tetraodon*, medaka and zebrafish), we conducted positive selection analyses (see Methods for details). We identified a set of 1067 genes that are evolving noticeably faster in the sunfish lineage compared with other branches (branch model, Additional file 2). In addition, using the branch-site model, we identified 1117 genes that contain positively selected sites specifically in sunfish (Additional file 3). We examined genes involved in the growth pathway and found several fast-evolving genes and genes containing positively selected sites in the GH/IGF1 axis. Previous studies have shown that this axis has a crucial role in regulating the growth of the fish body [17, 18]. Given the massive body size and extraordinary growth rate of the sunfish, we analyzed these growth-related genes in more detail. The GH/IGF1 axis comprises several components - the insulin-like growth factors (IGFs), IGF receptors (IGFRs), IGF-binding proteins (IGFBPs), growth hormone (GH), growth hormone receptor (GHR) and the insulin receptor (INSR). GH is released by the pituitary whereas IGF-1 is released by the liver as a result of GH stimulation. GH and IGF-1 exert their effects through GHR, IGFR and INSR to modify cell growth and proliferation (Fig. 2). The overall result is increased growth and decreased differentiation or suppression of apoptosis [19, 20]. Using the branch models in Phylogenetic Analysis by Maximum Likelihood (PAML) [21], we found multiple genes that are evolving at a different rate from the rest of the tree. Among these, several genes in the GH/IGF1 axis (*ghr1*, *igf1ra*, *ifg1rb*, *grb2*, *akt3*, *irs2a* and *jak2a*) were found to be evolving rapidly in the sunfish lineage (*dN/dS* values higher than the background) (Fig. 2 and Additional file 1: Table S7).

We found that both copies of *igf1r* (*igf1ra* and *igf1rb*) contain positively selected sites in the sunfish (Fig. 2 and Additional file 1: Table S7). Interestingly, some of these positively selected sites are located within functional domains of these receptors. For example, positively selected sites within Igf1ra were located within the fibronectin type III domain (FnIII, Pfam identifier PF00041) and receptor L domain (RL, PF01030) (Fig. 2). Previous studies have shown that the FnIII domain is important for ligand binding [22, 23], thus mutations in this domain could change the affinity between IGF1R and its ligand. Although the RL domain does not directly bind ligands, many of the determinants responsible for hormone binding and ligand specificity map to this central site [24]. Conversely, Igf1rb contains three positively selected sites located within the furin-like cysteine-rich region (CR, PF00757) and two sites in the protein tyrosine kinase domain (TK, PF07714) (Fig. 2). These two domains are involved in receptor aggregation [25] and other functions such as enzyme activity, subcellular localization and interactions with other molecules [26]. Interestingly, mutations in the ligand-binding domain and the RL domain of IGF1R can result in growth retardation in humans [27]. Thus, positive selection of sites within these domains of Igf1ra and Igf1rb in the sunfish may have enhanced the function of these genes. We also found evidence for positively selected sites in the insulin receptor gene (*insr*), which is known to bind to IGF-1 and promote growth [28] (Fig. 2). Mutations in human INSR are known to cause Donohue syndrome, which is characterized by stunted growth [29]. A positively selected site (R457) in the sunfish Insr maps to a mutation (K487E) in the human INSR associated with Donohue syndrome.

**Fig. 3:**
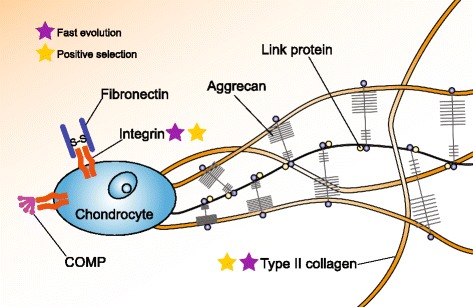
Genes related to bone and cartilage. Schematic diagram showing the extracellular matrix of cartilage (adapted from [82]). The figure illustrates collagens (mostly type II collagen), proteoglycans (primarily aggrecan), and other non-collagenous proteins including link protein (yellow circles) and fibronectin. Stars denote fast evolution or positive selection. COMP, cartilage oligomeric matrix protein

Another interesting set of fast-evolving genes/genes with positively selected sites are those related to the ECM. The ECM provides the microenvironment of the cell as well as bulk, shape and strength to tissues such as bone and cartilage. In addition, the ECM also contains components required for the conversion of cartilage to bone and its homeostasis [30, 31]. We found a number of genes related to the ECM that exhibit fast evolution or positive selection (Additional file 1: Table S8). The gene *COL2A1* encoding type II collagen, which normally represents approximately 80–90 % of the collagen content of the cartilage matrix [32] (Fig. 3), is present in two copies (*col2a1a* and *col2a1b*) and contains positively selected sites in the sunfish (Additional file 1: Table S8). Several ECM-related genes (*col11a1a, col11a2, bmp1b, fkbp10b*, *lepre1, serpinf1* and *sp7*) also exhibit elevated *dN/dS* values (Additional file 1: Table S8) but as there are no signs of positive selection, these genes might be under relaxed selection.

### Genes involved in bone formation

In contrast to other bony fishes (Osteichthyes), the endoskeleton of the sunfish is mainly composed of cartilage [5]. The genetic mechanism underlying this derived phenotype is not known. To look for clues to this mechanism, we analyzed genes known to be involved in bone formation such as those encoding proteoglycans, the bone morphogenetic protein (BMP) signalling pathway, transcription factors, bone differentiation and secretory calcium-binding phosphoproteins (SCPP). However, the sunfish possesses intact orthologues for most of these genes except for some SCPP genes (see Additional file 4). The details of the searches and the genes identified are given below.

#### Proteoglycan-encoding genes

Genes encoding proteoglycans are important regulators of cartilage and bone formation. We searched for the small leucine-rich proteoglycan (SLRP) gene family clusters: (a) *Fmod, Prelp, Optc*; (b) *Ecm2, Aspn, Omd, Ogn*; (c) *Dcn, Lum, Kera, Epyc*; and (d) *Ecm2-like* and *Bgn*. We first ran BLASTP (with default settings) of the human, zebrafish and/or fugu reference proteins against the annotated sunfish proteins. For genes that could not be identified using this method, we proceeded to a TBLASTN of the human and fish proteins against the sunfish genome assembly followed by a BLASTX of the resulting sunfish genomic loci against the NCBI non-redundant (NR) protein database. Using this strategy, we identified orthologues for all the above genes in the sunfish genome on (a) scaffold10.1, (b) scaffold39.1, (c) scaffold20.1, and (d) scaffold77.1, except *Optc* and *Omd*. In addition, we identified second copies of *Ogn* and *Fmod* located within nine genes of each other on scaffold13.1 (Additional file 5). We BLASTX-searched (with default settings) the sunfish loci of (a) and (b) against the NCBI NR protein database to identify *Optc* and *Omd* respectively, but did not identify these genes. *Optc* and *Omd* are present in zebrafish and cavefish but are absent in the Percomorphaceae fishes such as fugu, *Tetraodon*, tilapia and platyfish. Thus, they may not be responsible for the cartilaginous skeleton of the sunfish. We also searched for the lectican-hyaluronan- and proteoglycan-binding link protein (HAPLN) gene family clusters (*Hapln2* and *Bcan*; *Vcan* and *Hapln1*; *Acan* and *Hapln3*; *Ncan* and *Hapln4*) and other non-clustered proteoglycans (*Fn1*, *Lepre1, Tuft1, Podn*). Orthologues for all these genes are present in the sunfish (Additional file 4).

#### The BMP signalling pathway

We identified homologues for *Bmp2, Bmp4, Bmp5, Bmp6* and *Bmp7*; the receptors *Bmpr1a, Bmpr1b,* and *Bmpr2*; the Smad family genes (*Smad1, Smad4, Smad5, Smad6, Smad7*) and the Smurf family genes (*Smurf1, Smurf2*) in the sunfish genome. For *Smad4*, we identified up to four copies in the sunfish (Additional file 4).

#### Transcription factors

We identified homologues for *Sp7/osterix, Bapx1/Nkx3-2, Pdlim7, Mitf, Nfatc1, Msx1, Msx2* (the zebrafish homologue is known as *msxd*), *Hand1* and *Hand2* in the sunfish genome (Additional file 4). Runx2 is a key transcription factor involved in bone formation. Knockout of *Runx2* in the mouse results in the formation of cartilaginous skeleton [33]. The sunfish *Runx2* gene is located on scaffold14.1:1,387,125..1,422,518 (SUNFISH_GLEAN_10009694, see Additional file 4; the first 45 amino acids were removed because they are not present in other fish Runx2 proteins). An alignment of Runx2 proteins shows that the sunfish Runx2 is highly conserved (e.g. its DNA-binding domain is perfectly conserved and its central and C-terminal domains also look intact) (Additional file 1: Figure S2).

#### Bone differentiation genes

We searched for genes responsible for bone differentiation in the sunfish genome. We identified *leptin* and *leptin receptor, Bglap/osteocalcin, Fam20c, Bmp1, osteocrin, osteopotentia homologue, sclerostin, Phospho1* and *Phospho2*. We did not find *Mmp1* in sunfish. *Mmp1* may have arisen through tandem duplications in tetrapods based on our observations from the Genomicus database (Ensembl release 76). For *osteocrin*, we identified a partial prediction (one exon only) on scaffold56.1:442 kb between the genes *Gmnc* and *Iws1*, which are linked to *osteocrin* in the tilapia genome (on LG14). Thus, sunfish seems to have all the important genes involved in bone differentiation in teleost fishes (Additional file 4).

#### SCPP gene family

The SCPP gene family encodes secretory calcium-binding phosphoproteins that participate in the mineralization of collagenous bone and dentin as well as noncollagenous enamel. The SCPP genes originated from tandem duplication of the secreted protein acidic and rich in cysteine (SPARC)-like 1 gene (*Sparcl1*) that was itself derived from *SPARC* around the time a mineralized skeleton arose in vertebrates [34]. *SCPP* genes encode ECM proteins and are divided into two categories: acidic and proline/glutamine-rich (P/Q-rich). Whereas acidic SCPP genes are involved in the mineralization of collagenous bone and dentin, P/Q-rich SCPP genes participate in the deposition of non-collagenous enamel [34]. The elephant shark, which is a cartilaginous fish (Chondrichthyes) and lacks endochondral bone, does not contain any SCPP genes [35], whereas bony vertebrates contain both categories of SCPP genes although the complement of each category varies between lineages due to lineage-specific expansion and losses [36].

**Fig. 4:**
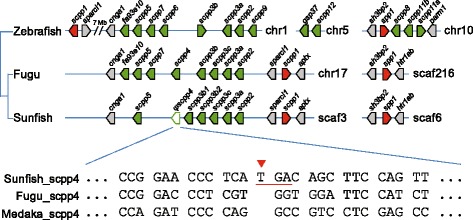
Scpp genes in the ocean sunfish. Upper panel: Comparison of the sunfish *sparcl1* locus with those of zebrafish and fugu showing the missing Scpp genes in sunfish (*fa93e10*, *scpp7* and *scpp4*). The *scpp4* pseudogene in the ocean sunfish is shown as a dotted arrow. Lower panel: Alignment of sunfish, fugu and medaka *scpp4* sequences showing the single base insertion in exon 2 of this gene in sunfish resulting in a premature termination codon followed by a frameshift in the rest of the open reading frame. This insertion was confirmed by PCR and sequencing of genomic DNA from two other specimens (GenBank accession numbers KF737069 and KF737070). The termination codon in sunfish is underlined in red. The accession numbers for fugu and medaka *scpp4* genes used in the alignment are DQ066525.1 and XM_004065875.2, respectively. chr, chromosome; scaf, scaffold

We searched for SCPP genes in the sunfish genome to understand the genetic basis of the cartilaginous skeleton of the sunfish. We filled a sequencing gap in this intergenic region by sequencing a genomic PCR product to obtain the complete sequence for *spp1*. The orthology of the P/Q-rich SCPP genes in sunfish was verified by generating a Maximum Likelihood phylogenetic tree using sequences from sunfish, fugu, medaka and zebrafish (see Additional file 1: Figure S3). Sunfish contains two acidic SCPP genes (*spp1* and *scpp1*) similar to fugu and zebrafish. However, it has lost two P/Q-rich SCPP genes (*fa93e10* and *scpp7*) that are conserved in the other two teleosts (Fig. 4 and Additional file 4). This conclusion was reached after searching the genomic vicinity of the sunfish *Sparcl1*, the entire genome assembly and the raw reads of the sunfish genome using zebrafish fa93e10 and scpp7 protein sequences by TBLASTN. *fa93e10* was first identified as an expressed sequence tag (EST) clone as part of a screen for genes that are expressed in regenerating zebrafish caudal fins [37]. Whole-mount in situ hybridization experiments in zebrafish suggested that *fa93e10* is a growth marker that identifies cycles of growth in fin ray segments [37]. Its frequency of expression in fin rays decreases with the age of the fish in tandem with decreased distal mesenchymal cell proliferation [37, 38]. It is not clear whether the absence of *fa93e10* in sunfish is somehow related to its unusual fin morphology. In addition to the complete loss of *fa93e10* and *scpp7*, another P/Q-rich SCPP gene, *scpp4*, that is intact in fugu and medaka has become a pseudogene in the sunfish due to a single nucleotide insertion. This insertion was confirmed by PCR and sequencing of genomic DNA from two other unrelated specimens of sunfish (GenBank accession numbers KF737069 and KF737070), providing further evidence that *scpp4* became nonfunctional in the sunfish lineage after it split from the pufferfish lineage (Fig. 4 and Additional files 1 and 4). However, the functional consequence of the loss of this gene in sunfish is not known. Zebrafish does not contain an orthologue of *scpp4* but instead contains several other lineage-specific P/Q-rich SCPP genes (Fig. 4 and Additional file 4). Thus, the genetic basis of the cartilaginous skeleton in the sunfish remains unclear. It is possible that this distinctive phenotype is driven by a regulatory change and is therefore not evident in the bone gene repertoire.

### Hox genes

Hox genes specify segmental identities along the anterior-posterior axis of developing vertebrate embryos and are also important for the anterio-posterior and proximal-distal patterning of limbs [39, 40]. Loss of Hox gene function can lead to morphological changes [41, 42]. Therefore, we hypothesized that loss of Hox genes could contribute to phenotypic evolution. To determine whether sunfish has uniquely lost any Hox gene(s), we analyzed the Hox gene complement in the sunfish. The sunfish possesses seven Hox gene clusters similar to fugu but contains more Hox genes (47 genes in sunfish compared with 45 in fugu) (Additional file 1: Figure S4). It possesses an intact *hoxa7a* and *hoxb7a* that are pseudogenes in fugu, making fugu the only known teleost completely lacking a Hox7 paralogous group member. In addition, sunfish has an intact *hoxc1a*, which is a pseudogene in fugu but is completely lost in medaka (Additional file 1: Figure S4). Conversely, sunfish lacks *hoxd11b*, which is present in fugu. However, this gene is also lost in fishes such as medaka and the East African cichlid (Additional file 1: Figure S4) [43]. Thus, it is unclear whether the loss of this gene has any effect on the overall morphology of sunfish. As such, the unusual morphology of the sunfish does not seem to be related to the loss of any specific clustered Hox gene. However, we cannot rule out the possibility of altered expression pattern(s) of Hox genes contributing to this unusual phenotype. In fact, the loss of the pelvic fin in fugu has been shown to be associated with the altered expression pattern of *Hoxd9a* [44].

## Discussion

Given its large size, fast growth rate and unusual body shape, the sunfish has received considerable attention from the research community. However, the genetic mechanisms underlying this ‘natural mutant’ have not been explored. The reference genome of the sunfish presented in this study provides first insights into the genomic changes that have occurred in this natural mutant. A major finding of our study is the identification of fast evolving and positively selected genes that are associated with growth in vertebrates. Although growth factors such as GH and IGF-1 do not show marked changes, several crucial receptor genes (*ghr1*, *igf1ra/b* in the GH/IGF1 axis) were found to have experienced positive selection or fast evolution. In addition, we found evidence for fast evolution and/or positive selection in some downstream genes in the GH/IGF1 axis (*irs2a*, *akt3*, *grb2* and *jak2a*), indicating that the GH/IGF1 axis might have played a crucial role in the large body size and rapid growth rate of the sunfish.

Despite being a member of the bony fish clade, the sunfish possesses a degenerate and cartilaginous skeleton. We found that a number of genes involved in the ECM, especially the genes encoding collagen, have undergone positive selection or fast evolution. In particular, several collagen genes involved in cartilage formation were found to be positively selected, which might have contributed to the predominantly cartilaginous skeleton of this gigantic fish. In addition, it has been demonstrated that the GH/IGF1 axis also has important roles in the regulation of bone and cartilage development [45]. We suspect that molecular changes in the GH/IGF1 axis unravelled in this study could also have affected the evolution of skeletal structures in the sunfish. We did not find any definitive molecular signatures related to the loss of the caudal fin in the sunfish. One reason could be that the molecular changes responsible for this phenotype could have occurred mainly in *cis*-regulatory elements.

## Conclusions

In summary, our analyses provided first insights into the molecular basis of the fast growth rate and large size of the sunfish. It also provides some clues to the genetic basis of the predominantly cartilaginous skeleton of this teleost fish. The high-quality genome assembly generated in this study should facilitate future studies of adaptations and population genetics of this enigmatic fish.

## Methods

### Genome sequencing and assembly

The sunfish blood sample was collected in 1998 from a sunfish stranded in an intra-coastal waterway in Florida, USA and stored frozen at −80 °C. The frozen blood was transported to Singapore in dry ice. Genomic DNA was extracted using the standard phenol/chloroform extraction method, suspended in TE buffer, and stored at 4 °C. We constructed eight paired-end libraries with insert sizes of 170 base pairs (bp), 500 bp, 800 bp, 2 kb, 5 kb, 10 kb, 20 kb and 40 kb and generated a total of 98.22 Gb sequence data (Additional file 1: Table S1, NCBI Project number PRJNA305960, SRA number SRA319445) on an Illumina HiSeq 2000 platform. We used SOAPdenovo 2 [46] to assemble the genome using K = 23 (map_len = 32 for libraries with shorter than 1 kb inserts, and map_len = 35 for libraries with inserts longer than 1 kb). Reads with low-quality bases (i.e., reads with more than 40 bases with quality scores less than 8 (Phred + 64), or containing more than ten Ns) or potential sequencing errors were removed or corrected by k-mer frequency-based methodology. After these quality control and filtering steps, a total of 68.87 Gb (96-fold coverage) clean data were retained for the sunfish assembly (Additional file 1: Table S2). The total length of the assembled genome is 642 Mb (Additional file 1: Table S4). To assess the assembly quality, we used the Core Eukaryotic Genes Mapping Approach (CEGMA) [47] and found that 99.6 % of the CEGMA genes are complete in the assembly. Analysis of Benchmarking Universal Single-Copy Orthologs (BUSCO) genes [48] showed that the assembly contains 74 % complete and 18 % partial vertebrate BUSCO orthologues. These results suggest that the assembly is of high quality. The genome assembly of the sunfish is available at DDBJ/ENA/GenBank under the accession number MBDK00000000.

### Estimation of genome size using k-mer analysis

We estimated the genome size using the k-mer method [49] and the formula: G = k-mer_number/k-mer_depth, where the k-mer_number is the total number of k-mers, and k-mer_depth denotes the peak frequency that occurred more than any other frequencies. For sunfish, the k-mer size was 17, k-mer_number was 20,842,591,260 and the k-mer_depth was 28, so the sunfish genome size was estimated to be 730,752,424 bp (Additional file 1: Table S3 and Figure S5).

### Annotation

We employed Tandem Repeats Finder (TRF) [50] to identify tandem repeats. RepeatMasker [51] and RepeatProteinMask [51] were used to identify and classify transposable elements (TEs) by aligning the sunfish genome sequences against a library of known repeats, Repbase [52], using default parameters. RepeatModeler [53] was used for *de novo* identification of repeats. All repeats obtained by various methods were combined together to form a non-redundant list of sunfish repeats (Additional file 1: Tables S5 and S6). For comparison, we also predicted repetitive sequences in other fishes using the same method (data shown in Fig. 1).

We used both homology-based and *de novo* methods for predicting protein-coding genes. For homology prediction, protein sets of human, fugu, *Tetraodon*, medaka and zebrafish were downloaded from Ensembl (release 73) [54] and mapped onto the sunfish genome using TBLASTN (v2.2.19) [55] (with E-value threshold of 1e-5). Genewise (with alignment rate threshold of 0.25) was used to generate gene models. For *de novo* prediction, Augustus (v2.5.5) [56] with the following parameters was used: uniqueGeneId = true; noInFrameStop = true; gff3 = on; strand = both, with gene model parameters trained on 1000 high-quality sunfish genes from homology-based predictions. These 1000 high-quality genes have intact gene models and span more than 90 % of the TBLASTN alignment. Finally, homology-based and *de novo* gene models were merged to form a comprehensive and non-redundant reference gene set using GLEAN [57] with the following parameters: minimum coding sequence length 150 bp and maximum intron length 10 kb.

### Gene family clustering

We used TreeFam [58] to cluster gene families in five fish genomes (sunfish, fugu, *Tetraodon*, medaka and zebrafish). We set maximum gene family size as 250 and BLAST E-value cutoff as 1e-7. In total, we obtained 14,768 gene families (Fig. 1). Sunfish-specific genes are the genes in gene families that exist only in sunfish.

### Phylogenetic reconstruction and divergence time estimation

The orthologous genes of seven ray-finned fishes (fugu, FUGU4; *Tetraodon*, TETRAODON8; stickleback, BROADS1; medaka, MEDAKA1; tilapia, Orenil1.0; zebrafish, Zv9 and spotted gar, LepOcu1) were acquired from Ensembl (release 76) [59]. In this analysis, we used fugu as the query (each gene at > 70 % identity with the fugu gene). The Ensembl dataset was further filtered to retain only one-to-one orthologues from all seven species. Inparanoid (v4.1) [60] was used to identify one-to-one orthologues in the sunfish using fugu for comparison (using default settings, i.e. minimum 50 % alignment span, minimum 25 % alignment coverage, minimum BLASTP score of 40 bits, minimum inparalog confidence level of 0.05, and score exceeding 300). Both the Ensembl and Inparanoid datasets were then combined, to obtain the final dataset of 1690 one-to-one ray-finned fish orthologues (Additional file 5). Their protein sequences were aligned using Clustal Omega (v1.2.0) [61]. Coding sequence alignments were generated based on the protein alignments using PAL2NAL (v14) [62]. Concatenated alignments were prepared for both the protein and coding sequence alignments by concatenating alignments of all orthologues. Gaps were removed from both the concatenated alignments using Gblocks (v0.91b) [63] at default settings. The lengths of the trimmed protein and coding sequence alignments were 888,142 and 2,705,059 positions, respectively.

For phylogenetic analyses, we used the Maximum Likelihood (ML) method. ModelGenerator (v0.85) [64] was used to obtain the best-fit substitution model for each concatenated alignment. The general time reversible (GTR) model with optimization of substitution rates and Gamma model of rate heterogeneity (GTR + I + G) and JTT (Jones-Taylor-Thornton) with an estimate of proportion of invariable sites, and Gamma model of rate heterogeneity and empirical base frequencies (JTT + I + G + F) were the selected models for the coding sequence and protein alignments, respectively. We used the rapid bootstrap algorithm with a thorough ML search (‘-f a’ option) as implemented in RAxML (v8.0.26) [65] and 100 bootstrap replicates to generate the trees.

The sequences of 1690 single-copy gene families from eight fishes (as described above) were concatenated and preprocessed for estimating divergence times based on the topology obtained in the phylogenomic analysis. MCMCTREE [9] (PAML package) was used to infer divergence times based on the approximate likelihood calculation method. Two nodes were calibrated using fossil records [66] as follows: ancestral node of fugu and *Tetraodon*: 32.25 to 56.00 mya; ancestral node of zebrafish and medaka; 149.85 to 165.20 mya.

### SNP calling and heterozygosity estimation

We used reads from 500 bp insert-size library to call SNPs. Firstly, we mapped sequencing reads to the assembled genome by the Burrows-Wheeler Alignment (BWA) tool [67] with the parameter ‘-n 4’. The average coverage depth of mapped reads was about 26 and covered about 98 % of the genome. We employed the Picard-tools [68] to prepare and filter alignments for SNP calling, including removing low-quality alignments, sorting alignments and merging duplication reads. Next, we used the Genome Analysis Toolkit (GATK) [69] to realign and recalibrate the bases that were disrupted by indel sites, and called SNPs. We filtered the SNPs that are next to another SNP within 10 bp and whose depth is less than five. The heterozygosity rate of sunfish was calculated as the number of heterozygous SNPs divided by effective genome size (genomic bases covered by at least five reads). We identified 489,862 heterozygous SNPs, and the heterozygosity rate of sunfish is 0.78 × 10^−3^.

### Population history estimation

The pairwise sequentially Markovian coalescent (PSMC) model is a coalescent-based hidden Markov model which can be used to estimate the history of effective population sizes based on genome-wide diploid sequence data [13]. Based on modelling of two sequences of the diploid genomes, this method can infer population size histories beyond 20,000 years. This method has been previously used for inferring demographic histories of vertebrates over a long evolutionary period [70–72]. Based on the result of SNP calling, we estimated the population size history of sunfish by PSMC [13]. To calculate the mutation rate required, we generated LASTZ [73] pairwise alignments of sunfish, *Tetraodon* and medaka. Based on the pairwise alignments, the difference per site between sunfish and *Tetraodon* is 0.2255, 0.2425 for sunfish and medaka, and 0.3244 for *Tetraodon* and medaka. The accumulated mutations in sunfish for the nearest common ancestor (NCA) of *Tetraodon* and sunfish to present is (0.2255 + 0.2425 - 0.3244) / 2 = 0.0718, and the site mutation rate of sunfish is 0.0718 / 68,000,000 = ~1 × 10^−9^ per site per year. In addition, we set the average generation time of sunfish as 4 years, as this is its medium population doubling time [74]. We performed PSMC with the parameters of the maximum 2 N0 coalescent time of 15, the maximum number of iterations of 30 and the pattern of parameters of ‘4 + 10 * 1 + 20 * 2 + 4 + 6’. We also performed 100 rounds of bootstrapping with the same parameters. We combined all the results and plotted the figure using the plot tool in PSMC (Additional file 1: Figure S1).

### Fast-evolving genes and genes with positively selected sites

To perform the *dN/dS* analysis, we generated a new set of orthologues using the gene sets of sunfish, fugu, *Tetraodon*, medaka and zebrafish. We used BLAST to obtain the reciprocal best hits (RBHs) for each pair of species (BLAST E-value cutoff of 1e-5). Finally, we identified 10,660 teleost homologues among these five species. We employed the software PRANK-MSA (v140110) [75] with the following parameters: gaprate = 0.025, gapext = 0.75, to generate coding sequence alignment for each orthologous group. We then used GUIDANCE (v1.41) [76] to mask nucleotides of low quality to Ns under the parameters of bootstraps = 10; seqCutoff = 0.6; colCutoff = 0.93. We regarded the sites with GUIDANCE site-wise score of < 0.5 as low-quality sites.

To examine the selective constraints on the genes, we estimated the *dN/dS* ratio (ω) using PAML (v4.4b) [21] with the coding sequence alignments obtained above. Firstly, we ran the branch models (mode = 2; NSsite = 0) [77] to estimate the ω across the tree ‘((sunfish, (fugu, tetraodon)), medaka, zebrafish);’ with the following parameter settings: Codonfreq = 2; kappa = 2.5; initial omega = 0.2. We used three hypotheses: 1) H0 hypothesis, all branches have the same ω; 2) H1 hypothesis, the branch leading to sunfish has a different ω whereas the other branches have the same ω; 3) H2 hypothesis, all branches have independent ω. We used likelihood values and degree of freedoms of the three hypotheses to perform a likelihood-ratio test (LRT). We picked up genes whose likelihood values of H1 are significantly larger (adjusted LRT *p*-value of < 0.05) than H0 and likelihood values of H2 are not significantly larger than H1. The genes with larger ω values in sunfish than other branches suggest their fast evolution in sunfish. Finally, we identified 1.067 fast-evolving genes with significant false discovery rate (FDR)-corrected *p*-values (<0.05) in the sunfish.

In addition, we also ran the branch-site models (model = 2; NSsite = 2) to detect the genes with positively selected sites in sunfish. For null hypothesis we set ‘fix_omega = 1; omega = 1’, whereas for the alternative hypothesis we set ‘fix_omega = 0; omega = 1.5’ with the tree ‘((sunfish #1, (fugu, tetraodon)), medaka, zebrafish)’. Using an FDR-corrected LRT *p*-value (adjusted LRT *p*-value) cutoff of 0.05, we identified 1117 positively selected genes in sunfish.

## Abbreviations

BLAST, basic local alignment search tool; BMP, bone morphogenetic proteins; ECM, extracellular matrix; GH/IGF1, growth hormone/insulin-like growth factor 1; INSR, insulin receptor; LRT, likelihood-ratio test; MPT, mid-Pleistocene climate transition; mya, million years ago; PSMC, pairwise sequentially Markovian coalescent; SCPP, secretory calcium-binding phosphoproteins; SNP, single nucleotide polymorphism

## Additional files

Additional file 1: Tables S1 to S9 and Figures S1 to S5. (DOCX 2557 kb) Additional file 2: Genes evolving significantly faster (S10a) or slower (S10b) in sunfish compared with fugu, *Tetraodon*, medaka and zebrafish (branch model). (XLSX 96 kb) Additional file 3: Genes containing positively selected sites in sunfish but not in fugu, *Tetraodon*, medaka and zebrafish (branch-site model). (XLSX 105 kb) Additional file 4: Presence/absence of bone formation-related genes in the sunfish genome. (XLSX 14 kb) Additional file 5: Gene IDs of 1690 one-to-one orthologues from the eight ray-finned fishes used for phylogenomic analysis and divergence time estimation. (XLSX 163 kb)

## References

[1] Pope E, Hays G, Thys T, Doyle T, Sims D, Queiroz N, Hobson V, Kubicek L, Houghton JR (2010). The biology and ecology of the ocean sunfish Mola mola: a review of current knowledge and future research perspectives. Rev Fish Biol Fish.

[2] Fraser-Brunner A (1951). The Ocean Sunfishes (Family Molidae), vol. 1.

[3] Powell DC (2001). A fascination for fish: adventures of an underwater pioneer.

[4] Nakatsubo T, Hirose H (2007). Growth of captive ocean sunfish, Mola mola. Suisan Zoshoku.

[5] Cleland J (1862). On the anatomy of the short sunfish (Orthragoriscus mola). Nat Hist Rev (Lond).

[6] Watanabe Y, Sato K (2008). Functional dorsoventral symmetry in relation to lift-based swimming in the ocean sunfish Mola mola. PLoS One.

[7] Nakae M, Sasaki K (2009). Peripheral nervous system of the ocean sunfish Mola mola (Tetraodontiformes: Molidae). Ichthyol Res.

[8] Luo R, Liu B, Xie Y, Li Z, Huang W, Yuan J (2012). SOAPdenovo2: an empirically improved memory-efficient short-read de novo assembler. Gigascience.

[9] Yang Z, Rannala B (2006). Bayesian estimation of species divergence times under a molecular clock using multiple fossil calibrations with soft bounds. Mol Biol Evol.

[10] Near TJ, Dornburg A, Eytan RI, Keck BP, Smith WL, Kuhn KL (2013). Phylogeny and tempo of diversification in the superradiation of spiny-rayed fishes. Proc Natl Acad Sci U S A.

[11] Arcila D, Alexander Pyron R, Tyler JC, Orti G, Betancur RR (2015). An evaluation of fossil tip-dating versus node-age calibrations in tetraodontiform fishes (Teleostei: Percomorphaceae). Mol Phylogenet Evol.

[12] Wu C, Zhang D, Kan M, Lv Z, Zhu A, Su Y (2014). The draft genome of the large yellow croaker reveals well-developed innate immunity. Nat Commun.

[13] Li H, Durbin R (2011). Inference of human population history from individual whole-genome sequences. Nature.

[14] Barash MS (2011). Environmental changes in the neogene and the biotic response. Oceanology.

[15] Hayward BW, Kawagata S, Grenfell HR, Sabaa AT, O’Neill T (2007). Last global extinction in the deep sea during the mid‐Pleistocene climate transition. Paleoceanography.

[16] Hayward BW (2002). Late Pliocene to Middle Pleistocene extinctions of deep-sea benthic foraminifera (“Stilostomella extinction”) in the southwest Pacific. J Foraminiferal Res.

[17] Fuentes EN, Valdes JA, Molina A, Bjornsson BT (2013). Regulation of skeletal muscle growth in fish by the growth hormone--insulin-like growth factor system. Gen Comp Endocrinol.

[18] Mommsen TP (2001). Paradigms of growth in fish. Comp Biochem Physiol B Biochem Mol Biol.

[19] Wood AW, Duan C, Bern HA (2005). Insulin-like growth factor signaling in fish. Int Rev Cytol.

[20] Brooks AJ, Waters MJ (2010). The growth hormone receptor: mechanism of activation and clinical implications. Nat Rev Endocrinol.

[21] Yang Z (1997). PAML: a program package for phylogenetic analysis by maximum likelihood. Comput Appl Biosci.

[22] Bass SH, Mulkerrin MG, Wells JA (1991). A systematic mutational analysis of hormone-binding determinants in the human growth hormone receptor. Proc Natl Acad Sci U S A.

[23] Clackson T, Wells JA (1995). A hot spot of binding energy in a hormone-receptor interface. Science.

[24] Garrett TP, McKern NM, Lou M, Frenkel MJ, Bentley JD, Lovrecz GO, Elleman TC, Cosgrove LJ, Ward CW (1998). Crystal structure of the first three domains of the type-1 insulin-like growth factor receptor. Nature.

[25] Raz E, Schejter ED, Shilo BZ (1991). Interallelic complementation among DER/flb alleles: implications for the mechanism of signal transduction by receptor-tyrosine kinases. Genetics.

[26] Radha V, Nambirajan S, Swarup G (1996). Association of Lyn tyrosine kinase with the nuclear matrix and cell-cycle-dependent changes in matrix-associated tyrosine kinase activity. Eur J Biochem.

[27] Abuzzahab MJ, Schneider A, Goddard A, Grigorescu F, Lautier C, Keller E (2003). IGF-I receptor mutations resulting in intrauterine and postnatal growth retardation. N Engl J Med.

[28] Lee J, Pilch PF (1994). The insulin receptor: structure, function, and signaling. Am J Physiol Cell Physiol.

[29] Ardon O, Procter M, Tvrdik T, Longo N, Mao R (2014). Sequencing analysis of insulin receptor defects and detection of two novel mutations in INSR gene. Mol Genet Metab Rep.

[30] Han L, Grodzinsky AJ, Ortiz C (2011). Nanomechanics of the Cartilage Extracellular Matrix. Annu Rev Mater Res.

[31] Velleman SG (2000). The role of the extracellular matrix in skeletal development. Poult Sci.

[32] Thomas JT, Ayad S, Grant ME (1994). Cartilage collagens: strategies for the study of their organisation and expression in the extracellular matrix. Ann Rheum Dis.

[33] Komori T, Yagi H, Nomura S, Yamaguchi A, Sasaki K, Deguchi K (1997). Targeted disruption of Cbfa1 results in a complete lack of bone formation owing to maturational arrest of osteoblasts. Cell.

[34] Kawasaki K (2011). The SCPP gene family and the complexity of hard tissues in vertebrates. Cells Tissues Organs.

[35] Venkatesh B, Lee AP, Ravi V, Maurya AK, Lian MM, Swann JB (2014). Elephant shark genome provides unique insights into gnathostome evolution. Nature.

[36] Kawasaki K (2009). The SCPP gene repertoire in bony vertebrates and graded differences in mineralized tissues. Dev Genes Evol.

[37] Goldsmith MI, Fisher S, Waterman R, Johnson SL (2003). Saltatory control of isometric growth in the zebrafish caudal fin is disrupted in long fin and rapunzel mutants. Dev Biol.

[38] Jain I, Stroka C, Yan J, Huang WM, Iovine MK (2007). Bone growth in zebrafish fins occurs via multiple pulses of cell proliferation. Dev Dyn.

[39] Zakany J, Duboule D (2007). The role of Hox genes during vertebrate limb development. Curr Opin Genet Dev.

[40] Alexander T, Nolte C, Krumlauf R (2009). Hox genes and segmentation of the hindbrain and axial skeleton. Annu Rev Cell Dev Biol.

[41] Fromental-Ramain C, Warot X, Messadecq N, LeMeur M, Dolle P, Chambon P (1996). Hoxa-13 and Hoxd-13 play a crucial role in the patterning of the limb autopod. Development.

[42] Swinehart IT, Schlientz AJ, Quintanilla CA, Mortlock DP, Wellik DM (2013). Hox11 genes are required for regional patterning and integration of muscle, tendon and bone. Development.

[43] Hoegg S, Boore JL, Kuehl JV, Meyer A (2007). Comparative phylogenomic analyses of teleost fish Hox gene clusters: lessons from the cichlid fish Astatotilapia burtoni. BMC Genomics.

[44] Tanaka M, Hale LA, Amores A, Yan YL, Cresko WA, Suzuki T, Postlethwait JH (2005). Developmental genetic basis for the evolution of pelvic fin loss in the pufferfish Takifugu rubripes. Dev Biol.

[45] Ohlsson C, Bengtsson BA, Isaksson OG, Andreassen TT, Slootweg MC (1998). Growth hormone and bone. Endocr Rev.

[46] Luo R, Liu B, Xie Y, Li Z, Huang W, Yuan J (2012). SOAPdenovo2: an empirically improved memory-efficient short-read de novo assembler. GigaScience.

[47] Parra G, Bradnam K, Korf I (2007). CEGMA: a pipeline to accurately annotate core genes in eukaryotic genomes. Bioinformatics.

[48] Simao FA, Waterhouse RM, Ioannidis P, Kriventseva EV, Zdobnov EM (2015). BUSCO: assessing genome assembly and annotation completeness with single-copy orthologs. Bioinformatics.

[49] Liu B, Shi Y, Yuan J, Hu X, Zhang H, Li N (2013). Estimation of genomic characteristics by analyzing k-mer frequency in de novo genome projects.

[50] Benson G (1999). Tandem repeats finder: a program to analyze DNA sequences. Nucleic Acids Res.

[51] Tarailo-Graovac M, Chen N (2009). Using RepeatMasker to identify repetitive elements in genomic sequences. Curr Protoc Bioinform.

[52] Bao W, Kojima KK, Kohany O (2015). Repbase Update, a database of repetitive elements in eukaryotic genomes. Mob DNA.

[53] Abrusán G, Grundmann N, DeMester L, Makalowski W (2009). TEclass—a tool for automated classification of unknown eukaryotic transposable elements. Bioinformatics.

[54] Flicek P, Ahmed I, Amode MR, Barrell D, Beal K, Brent S (2013). Ensembl 2013. Nucleic Acids Res.

[55] Kent WJ (2002). BLAT--the BLAST-like alignment tool. Genome Res.

[56] Stanke M, Waack S (2003). Gene prediction with a hidden Markov model and a new intron submodel. Bioinformatics.

[57] Elsik CG, Mackey AJ, Reese JT, Milshina NV, Roos DS, Weinstock GM (2007). Creating a honey bee consensus gene set. Genome Biol.

[58] Li H, Coghlan A, Ruan J, Coin LJ, Heriche JK, Osmotherly L (2006). TreeFam: a curated database of phylogenetic trees of animal gene families. Nucleic Acids Res.

[59] Kinsella RJ, Kahari A, Haider S, Zamora J, Proctor G, Spudich G (2011). Ensembl BioMarts: a hub for data retrieval across taxonomic space. Database.

[60] Remm M, Storm CE, Sonnhammer EL (2001). Automatic clustering of orthologs and in-paralogs from pairwise species comparisons. J Mol Biol.

[61] Sievers F, Higgins DG (2014). Clustal Omega, accurate alignment of very large numbers of sequences. Methods Mol Biol.

[62] Suyama M, Torrents D, Bork P (2006). PAL2NAL: robust conversion of protein sequence alignments into the corresponding codon alignments. Nucleic Acids Res.

[63] Castresana J (2000). Selection of conserved blocks from multiple alignments for their use in phylogenetic analysis. Mol Biol Evol.

[64] Keane TM, Creevey CJ, Pentony MM, Naughton TJ, McLnerney JO (2006). Assessment of methods for amino acid matrix selection and their use on empirical data shows that ad hoc assumptions for choice of matrix are not justified. BMC Evol Biol.

[65] Stamatakis A (2014). RAxML version 8: a tool for phylogenetic analysis and post-analysis of large phylogenies. Bioinformatics.

[66] Benton MJ, Donoghue PC (2007). Paleontological evidence to date the tree of life. Mol Biol Evol.

[67] Li H, Durbin R (2009). Fast and accurate short read alignment with Burrows–Wheeler transform. Bioinformatics.

[68] Picard Tools. http://broadinstitute.github.io/picard/. Accessed 30 March 2016.

[69] McKenna A, Hanna M, Banks E, Sivachenko A, Cibulskis K, Kernytsky A (2010). The Genome Analysis Toolkit: a MapReduce framework for analyzing next-generation DNA sequencing data. Genome Res.

[70] Liu Z, Liu S, Yao J, Bao L, Zhang J, Li Y (2016). The channel catfish genome sequence provides insights into the evolution of scale formation in teleosts. Nat Commun.

[71] Nadachowska-Brzyska K, Li C, Smeds L, Zhang G, Ellegren H (2015). Temporal Dynamics of Avian Populations during Pleistocene Revealed by Whole-Genome Sequences. Curr Biol.

[72] You X, Bian C, Zan Q, Xu X, Liu X, Chen J (2014). Mudskipper genomes provide insights into the terrestrial adaptation of amphibious fishes. Nat Commun.

[73] Harris RS. Improved pairwise alignment of genomic DNA. Ph.D. Thesis. The Pennsylvania State University; 2007.

[74] FishBase. http://www.fishbase.org. Accessed 30 March 2016.

[75] Loytynoja A (2014). Phylogeny-aware alignment with PRANK. Methods Mol Biol.

[76] Penn O, Privman E, Ashkenazy H, Landan G, Graur D, Pupko T (2010). GUIDANCE: a web server for assessing alignment confidence scores. Nucleic Acids Res.

[77] Zhao H, Yang JR, Xu H, Zhang J (2010). Pseudogenization of the umami taste receptor gene Tas1r1 in the giant panda coincided with its dietary switch to bamboo. Mol Biol Evol.

[78] Pan H, Yu H, Ravi V, Li C, Lee AP, Lian MM (2016). Genome of the ocean sunfish *(Mola mola*).

[79] Braasch I, Gehrke AR, Smith JJ, Kawasaki K, Manousaki T, Pasquier J (2016). The spotted gar genome illuminates vertebrate evolution and facilitates human-teleost comparisons. Nat Genet.

[80] Trobec K, von Haehling S, Anker SD, Lainscak M (2011). Growth hormone, insulin-like growth factor 1, and insulin signaling-a pharmacological target in body wasting and cachexia. J Cachexia Sarcopenia Muscle.

[81] Biasini M, Bienert S, Waterhouse A, Arnold K, Studer G, Schmidt T (2014). SWISS-MODEL: modelling protein tertiary and quaternary structure using evolutionary information. Nucleic Acids Res.

[82] Chen FH, Rousche KT, Tuan RS (2006). Technology Insight: adult stem cells in cartilage regeneration and tissue engineering. Nat Clin Pract Rheumatol.

