# Supplementary material for: The genome of the largest bony fish, ocean sunfish (Mola mola), provides insights into its fast growth rate
Source: Gigascience. 2016 Sep 9;5(1):36. doi: 10.1186/s13742-016-0144-3 (PMC5016917; doi:10.1186/s13742-016-0144-3)
Supplement: Additional file 1: Tables S1 to S9 and Figures S1 to S5. — (DOCX 2557 kb) [file 13742_2016_144_MOESM1_ESM.docx]

**Genome of the largest bony fish, ocean sunfish (*Mola mola*), provides insights into its fast growth rate**

**Additional file 1**

**Table S1.** **Statistics of the raw sequencing data**

| Pair-end libraries | Mate distance | Total data (Gb) | Read length (bp) | Sequence coverage (×) | Physical coverage (×) |
| --- | --- | --- | --- | --- | --- |
| Solexa Reads | 170 bp | 19.44 | 100 | 26.59 | 22.60 |
|  | 500 bp | 13.43 | 100 | 18.37 | 45.92 |
|  | 800 bp | 11.80 | 100 | 16.14 | 64.56 |
|  | 2 kb | 17.61 | 49 | 24.09 | 491.60 |
|  | 5 kb | 18.36 | 49 | 25.12 | 1,281.78 |
|  | 10 kb | 9.02 | 49 | 12.34 | 1,258.75 |
|  | 20 kb | 6.24 | 49 | 8.54 | 1,743.44 |
|  | 40 kb | 2.32 | 49 | 3.17 | 1,294.71 |
| Total | - | 98.22 | - | 134.36 | 6,203.36 |

**Table S2. Statistics of clean reads**

| Pair-end libraries | Mate distance | Total data (Gb) | Average read length (bp) | Sequence coverage (×) | Physical coverage (×) |
| --- | --- | --- | --- | --- | --- |
| Solexa Reads | 170 bp | 17.91 | 100 | 24.50 | 20.83 |
|  | 500 bp | 12.31 | 100 | 16.83 | 42.08 |
|  | 800 bp | 10.08 | 100 | 13.79 | 55.16 |
|  | 2 kb | 14.56 | 49 | 19.91 | 406.36 |
|  | 5 kb | 6.98 | 49 | 9.55 | 487.31 |
|  | 10 kb | 4.95 | 49 | 6.78 | 691.50 |
|  | 20 kb | 2.08 | 49 | 2.85 | 581.80 |
|  | 40 kb | 1.11 | 49 | 1.52 | 620.05 |
| Total | - | 68.87 | - | 95.73 | 2,905.09 |

Table S3. Genome size estimation based on 17-mer frequencies

| k-mer | k-mer num | pkdepth | Genome size | Used bases | Used reads | Coverage |
| --- | --- | --- | --- | --- | --- | --- |
| 17 | 20,461,067,886 | 28 | 730,752,424 | 25,675,655,900 | 302,066,540 | 34.5× |

The peak k-mer depth was 28 (see Figure S5); the total k-mer number was 20,461,067,886. The genome size can be calculated according to the formula: G = k-mer_num/k-mer_peak_depth.

**Table S4. Statistics of the ocean sunfish genome assembly**

|  | Contig | | Scaffold | |
| --- | --- | --- | --- | --- |
|  | Size (bp) | Number | Size (bp) | Number |
| N90 | 6,058 | 30,700 | 2,642,772 | 69 |
| N80 | 9,675 | 22,649 | 4,141,383 | 49 |
| N70 | 13,175 | 17,104 | 5,882,655 | 37 |
| N60 | 16,681 | 12,874 | 7,430,703 | 28 |
| N50 | 20,415 | 9,475 | 8,766,736 | 19 |
| Longest | 126,984 | ----- | 32,686,547 | ----- |
| Total Size | 627,913,194 | ----- | 641,653,604 | ----- |
| Total Number (> 100 bp) | ----- | 71,375 | ----- | 18,313 |
| Total Number (> 2 kb) | ----- | 43,301 | ----- | 480 |

## Table S5. Statistics of repeat annotation

| Species | Method | Repeat length | Genome length | % of genome length |
| --- | --- | --- | --- | --- |
| Ocean sunfish | RepeatProteinMask | 57,339,108 | 641,653,604 | 8.94% |
|  | RepeatMasker | 53,594,512 | 641,653,604 | 8.35% |
|  | TRF | 11,073,979 | 641,653,604 | 1.73% |
|  | Total (non-redundant) | 69,776,025 | 641,653,604 | 10.87% |
| Fugu | Total | 43,011,673 | 392,800,674 | 10.95 % |
| *Tetraodon* | Total | 34,337,732 | 358,618,246 | 9.58% |
| Medaka | Total | 79,948,019 | 869,000,216 | 9.20 % |
| Zebrafish | Total | 769,503,788 | 1,412,464,843 | 54.48% |

## The table shows results from three repeat annotation programs (ProteinMask, RepeatMasker and TRF), and the non-redundant total repeat sizes in the genomes of ocean sunfish, fugu, *Tetraodon*, medaka and zebrafish.

## Table S6. Composition of repetitive sequences in various teleosts

| **Type** | **% in genome** | | | | |
| --- | --- | --- | --- | --- | --- |
|  | **Ocean sunfish** | **Fugu** | **Medaka** | **Tilapia** | **Stickleback** |
| DNA | 2.1957 | 1.8842 | 3.3039 | 6.5297 | 1.5272 |
| LINE | 5.5932 | 3.4735 | 2.6143 | 7.5822 | 1.5930 |
| SINE | 0.5255 | 0.2232 | 0.6775 | 0.3553 | 0.0154 |
| LTR | 1.1212 | 1.0743 | 1.1432 | 4.3510 | 1.9645 |
| Other | 0.0001 | 0.0007 | 0.0002 | 0.0007 | 0.0001 |
| Satellite | 0.0001 | 0.0001 | 0.0000 | 0.0001 | 0.0001 |
| Simple_repeat | 0.7475 | 1.4466 | 0.2488 | 0.0391 | 0.0024 |
| Low complexity | 0.5732 | 0.4636 | 0.4521 | 0.4048 | 0.0004 |
| Tandem Repeat | 0.6515 | 2.1082 | 0.6107 | 0.4247 | 0.0009 |
| Unknown | 0.0848 | 0.2789 | 0.1457 | 0.0231 | 0.2976 |
| Total | 11.4929 | 10.9534 | 9.1964 | 19.7108 | 5.4014 |

**Table S7.** **Copy number and adjusted likelihood ratio test (LRT) p-values of selected sunfish genes in the growth hormone/insulin-like growth factor-1 (GH/IGF-1) axis**

| Gene | Branch-site model | Branch model | | |
| --- | --- | --- | --- | --- |
|  | Adjusted LRT p-value | Adjusted LRT p-value | *dN/dS* (sunfish) | *dN/dS* (background) |
| *ghr1* | 1.00E+00 | 2.24E-02 | 0.3503 | 0.219 |
| *igf1ra* | 3.64E-07 | 2.86E-02 | 0.1253 | 0.0893 |
| *igf1rb* | 2.30E-03 | 8.20E-03 | 0.1347 | 0.0883 |
| *insr* | 7.10E-03 | 2.72E-01 | 0.0789 | 0.0571 |
| *grb2* | 6.00E-02 | 9.40E-03 | 0.3926 | 0.018 |
| *akt3* | 7.50E-01 | 2.90E-08 | 0.0449 | 0.0105 |
| *irs1* | 4.99E-01 | 9.20E-03 | 0.0054 | 0.0658 |
| *irs2a* | 1.50E-03 | 3.40E-03 | 0.1532 | 0.0955 |
| *irs2b* | 2.71E-01 | 3.92E-03 | 0.0386 | 0.0873 |
| *jak2a* | 1.00E+00 | 1.15E-05 | 0.2009 | 0.0782 |
| *jak2b* | 7.49E-02 | 1.90E-03 | 0.0292 | 0.0851 |
| *stat5* | 9.98E-01 | 1.69E-02 | 0.0112 | 0.0263 |

Adjusted LRT p-values < 0.05 are shown in red font.

## Table S8. Copy number and adjusted likelihood ratio test (LRT) p-values of sunfish genes in the extracellular matrix (ECM)

| Gene | Branch-site model | Branch model | | |
| --- | --- | --- | --- | --- |
|  | Adjusted LRT  p-value | Adjusted LRT p-value | *dN/dS* (sunfish) | *dN/dS* (background) |
| *col1a1a* | 1.01E-12 | 8.51E-09 | 0.4692 | 0.1964 |
| *col1a1b* | 4.80E-08 | 6.66E-16 | 0.4923 | 0.1958 |
| *col2a1a* | 1.50E-04 | 4.60E-03 | 0.1593 | 0.0989 |
| *col2a1b* | 2.00E-03 | 2.00E-02 | 0.1476 | 0.1040 |
| *col1a2* | 6.00E-04 | 2.95E-09 | 0.6791 | 0.2573 |
| *col4a1* | 1.00E-04 | 4.20E-09 | 0.4024 | 0.1843 |
| *col4a2* | 0.00E+00 | 5.55E-16 | 0.6108 | 0.2130 |
| *col4a6* | 1.18E-12 | 1.35E-10 | 0.5783 | 0.2622 |
| *col11a1a (coba1a)* | 1.00E+00 | 3.54E-05 | 0.1840 | 0.0885 |
| *col11a2 (coba2)* | 1.00E+00 | 3.80E-02 | 0.1862 | 0.1372 |
| *col27a1a (cora1a)* | 3.71E-08 | 2.20E-03 | 0.3045 | 0.1876 |
| *itga1 (ita1)* | 1.58E-05 | 6.17E-01 | 0.1941 | 0.1784 |
| *itga10 (ita10)* | 1.00E+00 | 5.43E-02 | 0.1218 | 0.0944 |
| *itgb1a (itb1a)* | 3.70E-03 | 6.43E-01 | 0.0835 | 0.0897 |
| *itgb1b (itb1b)* | 7.39E-01 | 2.61E-02 | 0.0414 | 0.0754 |
| *itgb5 (itb5)* | 1.00E+00 | 4.54E-01 | 0.0795 | 0.0672 |
| *lamc1* | 8.93E-02 | 7.93E-01 | 0.0687 | 0.0659 |
| *thbs4a (tsp4a)* | 1.10E-02 | 8.69E-05 | 0.2272 | 0.0979 |
| *thbs4b (tsp4b)* | 1.06E-02 | 9.30E-03 | 0.0596 | 0.0950 |
| *bmp1a* | 6.04E-02 | 7.87E-01 | 0.0292 | 0.0310 |
| *bmp1b* | 1.00E+00 | 4.22E-05 | 0.0664 | 0.0333 |
| *Crtap* | 7.33E-02 | 2.01E-01 | 0.0947 | 0.0657 |
| *fkbp10a* | 2.20E-03 | 9.49E-01 | 0.1078 | 0.1063 |
| *fkbp10b* | 1.00E+00 | 1.30E-02 | 0.2114 | 0.1056 |
| *ifitm5* | 6.38E-01 | 9.10E-02 | 0.0979 | 0.0452 |
| *lepre1* | 7.01E-01 | 5.00E-04 | 0.2395 | 0.1203 |
| *Ppib* | 1.00E+00 | 7.78E-01 | 0.0693 | 0.0605 |
| *serpinf1* | 1.00E+00 | 1.23E-01 | 0.2141 | 0.1334 |
| *sp7* | 1.00E+00 | 1.61E-02 | 0.1422 | 0.0548 |

Adjusted LRT p-values < 0.05 are shown in red font.

**Table S9: *scpp4* PCR amplicon sequences obtained from two other sunfish samples that confirms the presence of a single base insertion in the coding sequence.**

>scpp4_PCR_amplicon sequence_Ocean sunfish sample KU # T2959

CTTACCTGCATTCACAAGGACAAGTGACTCAAGCAATGGGTCAGAAACCCAATGCTCAGACACGGACACCACTTTCCCCTCGGATGGAGCAACTGCAGCCTGGGGTCTTCCAGCAACCGGAACCCTCA**TGA**CAGCTTCCAGTTCCTGCCTTCCTCGCAACTCTATTCCTGGTCTCCACTAGGAGGCAGTCCTGTGTTTTTCCCTCTGCAGGTGGGCCTCCACACATTTTAGTCACTCCCACAGCAGCCTCTGCTCTGAAGGGATAAAGCACCTACATGCCAAAC

> scpp4_PCR_amplicon sequence_Ocean sunfish sample KU # T2979

CTTACCTGCATTCACAAGGACAAGTGACTCAAGCAATGGGTCAGAAACCCAATGCTCAGACACGGACACCACTTTCCCCTCGGATGGAGCAACTGCAGCCTGGGGTCTCCCAGCAACCGGAACCCTCA***T*GA**CAGCTTCCAGTTCCTGCCTTCCTCGCAACTCTATTCCTGGTCTCCACTAGGAGGCAGTCCTGTGTTTTTCCCTCTGCAGGTGGGCCTCCACACATTTTAGTCACTCCCACAGCAGCCTCTGCTCTGAAGGGATAAAGCACCTACATGCCAAAC

PCR primers used:

scpp4F: CTTACCTGCATTCACAAGGACAAG

scpp4R: GTTTGGCATGTAGGTGCTTTATCC


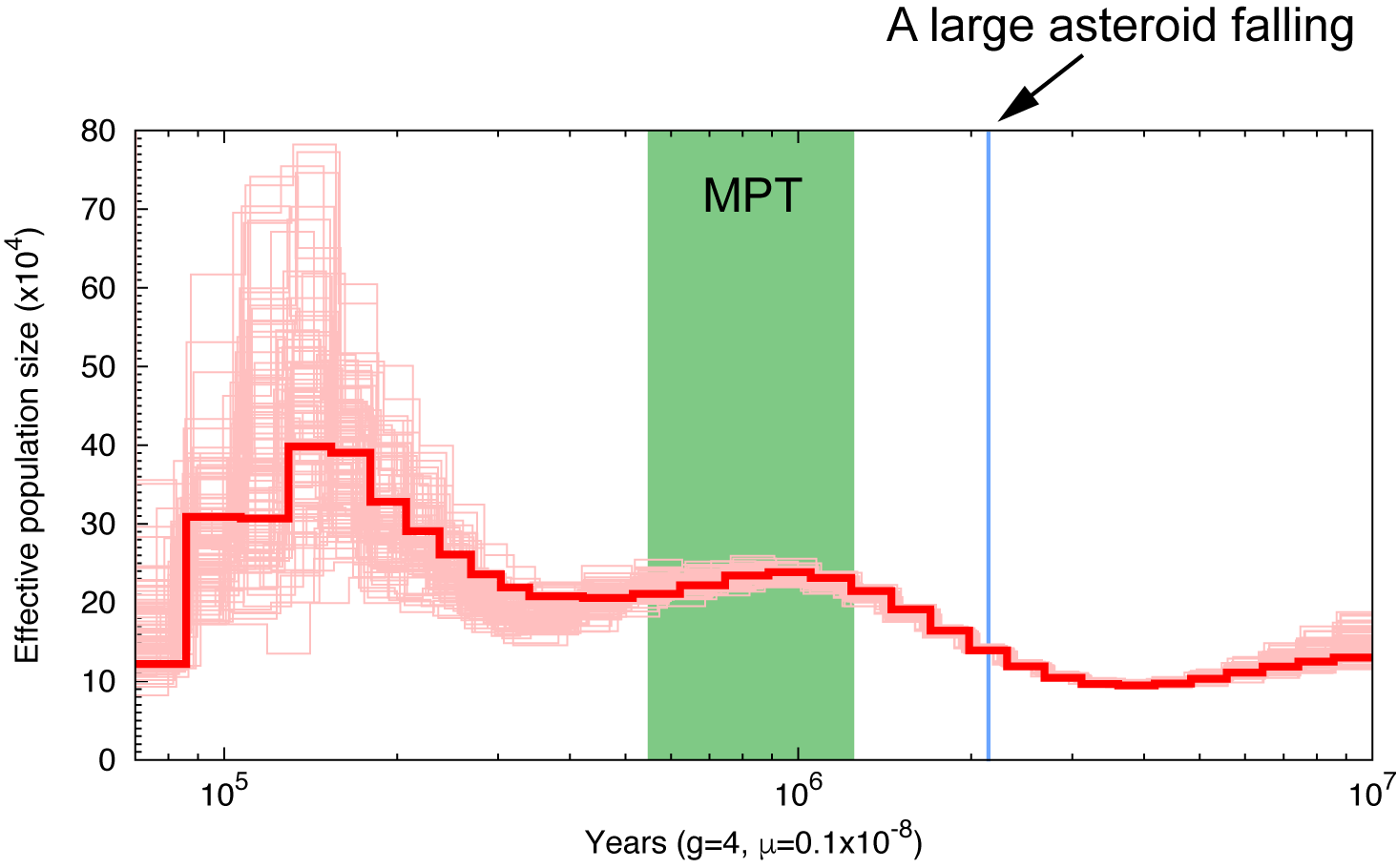


## Figure S1. The population history of ocean sunfish. The red line indicates changes in the estimated effective population sizes of ocean sunfish whereas the pink lines represent 100 bootstrap estimations. The green block represents the mid-Pleistocene climate transition period (MPT, ~1.2-0.55 million years ago (mya)). The blue line indicates the approximate time point when a large asteroid (> 1 km in diameter) fell and resulted in an extinction event (~2.15 mya).


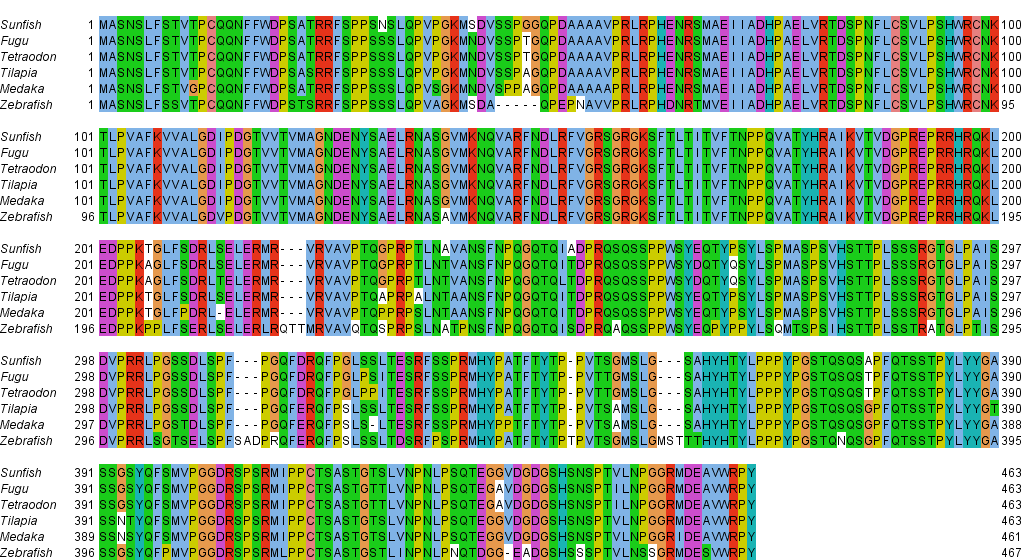


**Figure S2. Alignment of Runx2 proteins from six teleost fishes.** The alignment shows a high level of conservation between sunfish, fugu, *Tetraodon*, tilapia, medaka and zebrafish..

**
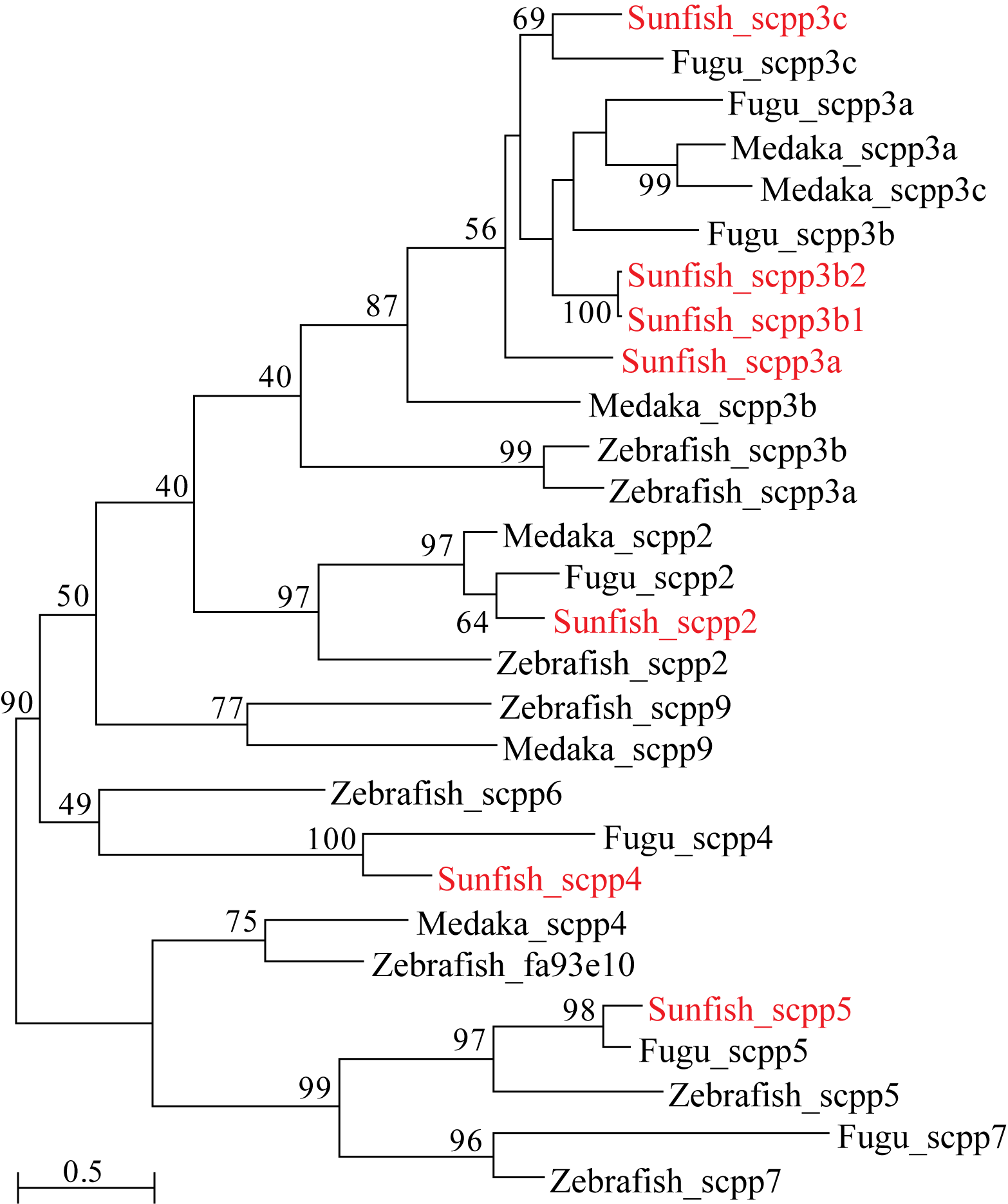
**

**Figure S3. Phylogenetic analysis of sunfish P/Q-rich SCPP genes.** A Maximum Likelihood (ML) tree of P/Q-rich SCPP genes from sunfish, fugu, medaka and zebrafish was generated using RAxML (ver. 8.1.3) [1] based on their amino-acid sequences to confirm the identities of the sunfish genes (in red font). The alignment was generated using Clustal Omega [2] and best-fit model (WAG+G+F) was deduced using ModelGenerator [3]. Values at the nodes represent bootstrap support percentages. Bootstrap values below 50 are not shown. The scale bar denotes number of substitutions per site.

**
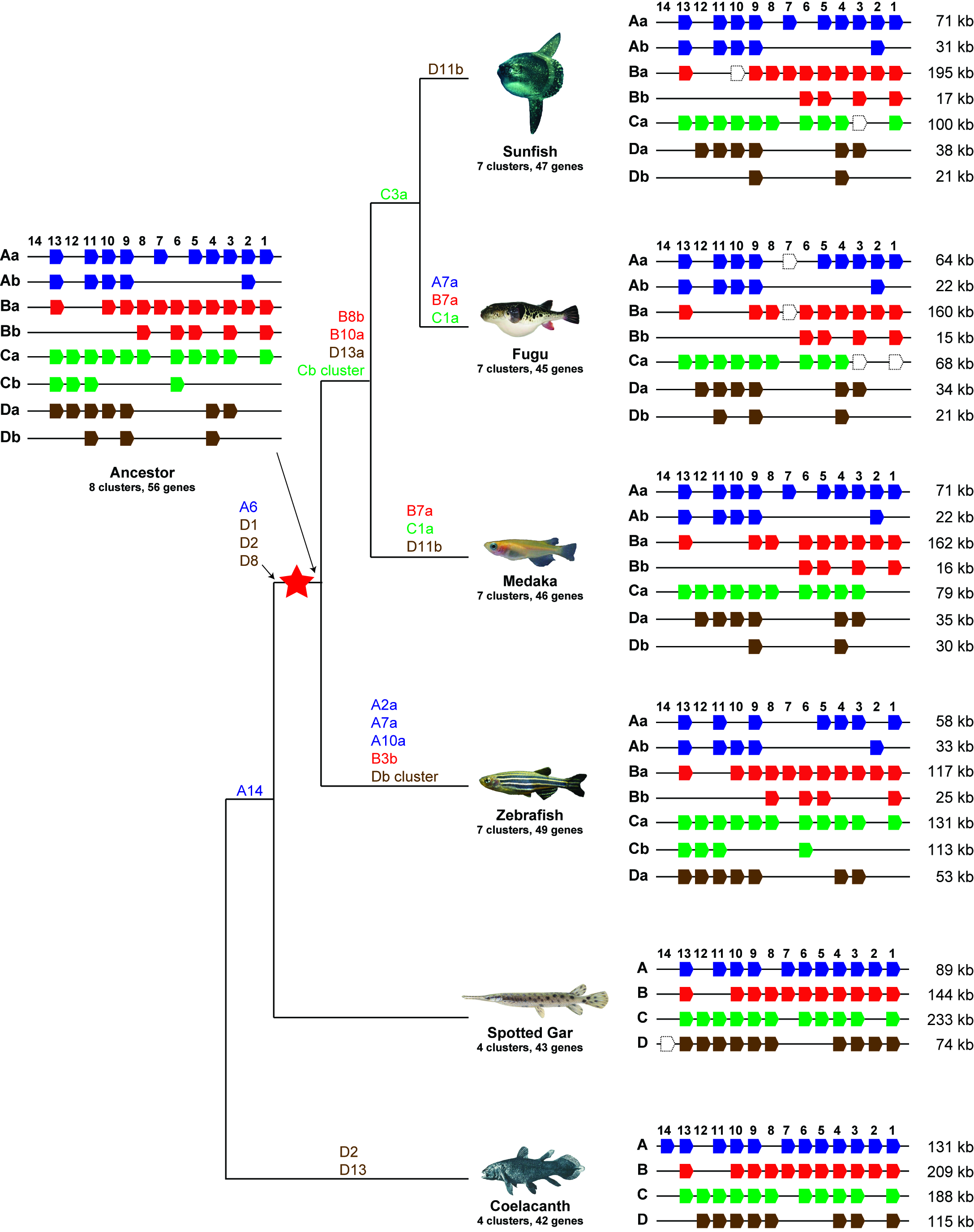
**

**Figure S4. Hox gene clusters in the ocean sunfish, selected teleosts, the spotted gar and the coelacanth.** Arrows represent Hox genes with the direction of the arrows representing transcriptional orientation. Dotted boxes represent pseudogenes. Hox genes/clusters lost are indicated above the ancestral as well as terminal branches of the tree. The star denotes the teleost-specific genome duplication event. Approximate sizes of the Hox clusters are indicated on the right. The ancestral teleost Hox cluster shown here is based on the Hox clusters of sunfish, fugu, medaka and zebrafish. Sunfish Hox Aa, Ab, Ba, Bb, Ca, Da and Db clusters are present on scaffolds 4.1, 41.1, 47.1, 36.1, 50.1, 7.1 and 11.1, respectively.

## Figure S5. 17-mer frequency curve of the ocean sunfish genome. The x-axis represents the depth coverage of each unique 17-mer in the genome, and the y-axis denotes the occurrence of unique 17-mers within the sequence dataset.

**References**

## 1. Stamatakis A. RAxML version 8: a tool for phylogenetic analysis and post-analysis of large phylogenies. Bioinformatics. 2014;30:1312-3.

## 2. Sievers F, Higgins DG. Clustal Omega, accurate alignment of very large numbers of sequences. Methods Mol Biol. 2014;1079:105-16.

## 3. Keane TM, Creevey CJ, Pentony MM, Naughton TJ, McInerney JO. Assessment of methods for amino acid matrix selection and their use on empirical data shows that ad hoc assumptions for choice of matrix are not justified. BMC Evol Biol. 2006;6:29.
